# UEG journal's editorial team

**DOI:** 10.1002/ueg2.12340

**Published:** 2022-11-24

**Authors:** Joost P. H. Drenth, Katarzyna M. Pawlak

**Affiliations:** ^1^ Department of Gastroenterology and Hepatology Radboudumc Nijmegen Nijmegen The Netherlands; ^2^ Endoscopy Unit Department of Gastroenterology Hospital of the Ministry of Interior and Administration Szczecin Poland

**Keywords:** editorial board, gastroenterology, trainee editors, UEG journal, visual abstracts

This special issue of the UEG Journal marks the end of an era for the Editorial board. As of 2023, we are parting with the current team of trainee editors. Just over 2 years ago, we selected, with the help of the UEG Young Talent Group, 10 great European talents. They have made all the difference for us. This team, amongst others consisting of Chloe Melchior, Diogo Libânio, Alberto Zanetto, Dragos Ciocan, and Alexandre Nuzzo, assisted us in the review process and have honed their skills in making razor‐sharp assessments when judging scientific work. They provided us with a different perspective and helped us to select those manuscripts that will eventually make a difference in your clinical practice. The activities of the trainee editors have rapidly expanded over the last 2 years. We expanded our presence on Social Media and amplified the message from the published material. For those who follow us on Twitter, you are not alone! Indeed, on Twitter, we have created a network with >8000 professionals who receive regular updates on the UEG Journal and our editorial work. If you decide to publish with us, we offer you a host of communication tools to get the message out. In the last issue we published on the benefits of search engine optimization, and Marcus Hollenbach and Willem‐Pieter Brouwer helped us to adapt title and abstract so that the paper is easily findable amongst the competitors. With the help of Livia Archibugi, we create a visual abstract of each published article that is used on social media. The start of 2023 will also be published on the Journal's website. Iago Rodriguez Lago and Katarzyna Pawlak spearhead the podcast initiative, and since its inception, they have interviewed 13 authors who co‐authored UEG Journal articles. The trainee editors have been very prolific and have contributed significantly to the UEG Journal over the last 2 years.^1^, ^2^, ^3^, ^4^, ^5^, ^6^, ^7^, ^8^, ^9^, ^10^, ^11^, ^12^, ^13^, ^14^, ^15^ We would like to pay tribute to these highly gifted colleagues who significantly improved the Journal by devoting their time to the UEG. The Editors have been privileged to have worked with you! As of 2023, new trainee editors will enter the publishing arena. Again the UEG Young talent group helped to make the cut and this time, **15 new talents** have been selected. We welcome them and look forward to seeing their impact on the Journal. You will also see some changes in the team of Associate Editors. After 4 years of service Hidekazu Suzuki and Deidre McNamara will leave us. They are wonderful colleagues and made great contributions to the Journal.^16^, ^17^, ^18^, ^19^ New to the Journal will be **Daniel Keszthelyi** and **Yasuko Maeda**. Daniel is a Gastroenterologist from Maastricht, The Netherlands, has experience in the field of Neurogastroenterology and Motility, and regularly contributes to the Journal.^20^, ^21^ Yasuko is a colorectal surgeon at Cumberland Infirmary and a member of the guideline committee of the European Society of Coloproctology, one of the specialist societies part of the UEG. She will be responsible for the guidelines that are offered and published in the UEG Journal.^22^ She is expected to work closely with UEG's Quality of Care task force chaired by Wafaa Khannoussi.^23^

UEG Journal continues further work under the Wiley® flag. This active, nearly 3‐year collaboration resulted in many improvements, including a smoother publishing process, increasing reach in the scientific market, introducing new marketing strategies, and upgrading the UEG Journal's website. We believe that all this, together with rigorously selected articles, contributed to an increase in Impact Factor that almost doubled (2017—3.543 to 2021—6.866)! Also, the UEG Journal visibility was boosted. Particular emphasis was put on engineering a way to share the journal's content in the most accessible form. Following trends, we wanted to send out a clear message: here you find top‐notch science, a can't‐miss. For this reason, the idea of UEG Journal brand‐specific visual abstracts was developed, and until now, almost 100 have already been published for selected articles. We are convinced that illustrations tell stories. That is why we introduced **Clinical image** to the UEG Journal as a new article type, providing you with exceptional pictures that capture a GI disease or clinical manifestation. More precisely, we hope to see the unusual visual presentations of a common disease, unexpected association between two relatively uncommon symptoms, or abrupt events in the course of managing a patient. Finally, UEG Journal is churning out guidelines thanks to the fine collaboration with the Quality of Care Task Force. Only in the last 2 years, the eight guidelines have been published covering functional and immunity disorders like achalasia, functional dyspepsia, gastroparesis, and microscopic colitis, but also diagnosis standards including carbon 13, hydrogen, and methane breath tests performance.^24^, ^25^, ^26^, ^27^, ^28^, ^29^

The future is bright for the UEG Journal thanks to its readers, contributors, editors, reviewers, and the UEG community!

## Data Availability

Data sharing is not applicable to this article as no new data were created or analyzed in this study.
